# Chronobiology Meets Quantum Biology: A New Paradigm Overlooking the Horizon?

**DOI:** 10.3389/fphys.2022.892582

**Published:** 2022-07-06

**Authors:** Gianluigi Mazzoccoli

**Affiliations:** Division of Internal Medicine and Chronobiology Laboratory, Department of Medical Sciences, Fondazione IRCCS “Casa Sollievo Della Sofferenza”, San Giovanni Rotondo (FG), Italy

**Keywords:** chronobiology, quantum biology, quantum mechanics, entanglement, tunneling, coherence, superposition, biological clock

## Abstract

Biological processes and physiological functions in living beings are featured by oscillations with a period of about 24 h (circadian) or cycle at the second and third harmonic (ultradian) of the basic frequency, driven by the biological clock. This molecular mechanism, common to all kingdoms of life, comprising animals, plants, fungi, bacteria, and protists, represents an undoubted adaptive advantage allowing anticipation of predictable changes in the environmental niche or of the interior *milieu.* Biological rhythms are the field of study of Chronobiology. In the last decade, growing evidence hints that molecular platforms holding up non-trivial quantum phenomena, including entanglement, coherence, superposition and tunnelling, *bona fide* evolved in biosystems. Quantum effects have been mainly implicated in processes related to electromagnetic radiation in the spectrum of visible light and ultraviolet rays, such as photosynthesis, photoreception, magnetoreception, DNA mutation, and not light related such as mitochondrial respiration and enzymatic activity. Quantum effects in biological systems are the field of study of Quantum Biology. Rhythmic changes at the level of gene expression, as well as protein quantity and subcellular distribution, confer temporal features to the molecular platform hosting electrochemical processes and non-trivial quantum phenomena. Precisely, a huge amount of molecules plying scaffold to quantum effects show rhythmic level fluctuations and this biophysical model implies that timescales of biomolecular dynamics could impinge on quantum mechanics biofunctional role. The study of quantum phenomena in biological cycles proposes a profitable “entanglement” between the areas of interest of these seemingly distant scientific disciplines to enlighten functional roles for quantum effects in rhythmic biosystems.

## Introduction

Biosystems are featured by multifrequency periodicity of electrochemical phenomena and biological processes. On planet Earth living organisms have become adapted to the sidereal period of Earth’s rotation on its axis (precisely 23 h 56 min and 4 s as for November 2021), dictating the alternation of sunlight and darkness and a solar day of 24 h on average. The intensity of natural lighting varies in relation to several factors: 1) incident light intensity at a geographical location naturally changes by approximately eight orders of magnitude during rotation of the Earth; 2) the distribution of relative darkness, counting twilight, moonlight, and starlight, shows striking seasonal variation due to the Earth’s orbital motion as well as axis tilt; and 3) the Moon’s orbit around the Earth and moonlight intensity set off regular changes in nighttime light environment (Gaston et al., 2014). Direct solar radiation, which reaches the ground without being disturbed by its path through the atmosphere, represents the energy source that makes life possible on planet Earth, which can be considered as a closed system comprising an enormous number of subsystems, including ecosystems. A unidirectional flow of energy passes through the ecosystems via processes that allow the transfer of energy and that transform light energy into biomass. This flow of energy that defines and governs the biotic component within the system persists in the trophic webs of ecosystems, in particular in the form of high-energy phosphate bonds available in biological systems which are a source of energy at the molecular level. The external environment represents a composite dimension in continuous modification and all living beings are in constant relationship with it through biological machineries. Many of the changes affecting the external environment occur with foreseeable rhythmicity over physical time intervals of variable duration and ranging from hours to years.

## Chronobiology

Chronobiology is the scientific discipline that studies the biological processes in relation to the chronological dimension and explores and quantifies size and set of periodic patterns of biotic phenomena in living beings. Rhythmicity with 24 ± 4 h swinging period is named “circadian” (from *circa dies*, i.e., roughly 1 day) a definition conceived by Franz Halberg in the early fifties (Halberg et al., 1955; Visscher and Halberg 1955). Cycles with time intervals below ∼24 h are defined “ultradian”, while cycles with time intervals above ∼24 h are defined “infradian”. Three properties hallmark circadian rhythms: 1) they persist (or free-run) with a period of ∼24 h in the absence of an external time cue (or *zeitgeber*); 2) retune according to modifications in environmental cues, most remarkably the everyday light/darkness alternation (entrained and phase shifted by light) and temperature cycles; and 3) time interval length does not vary over a wide range of physiologically applicable temperatures (temperature compensation). Living beings are capable to anticipate predictable environmental changes and in the wild carry out daily life activities, such as feeding, fasting, resting, hunting, migrating, and mating, at profitable times during the day/year, with distinguishing seasonal adjustments, through endogenous molecular clockworks that external signals, primarily light and temperature, can synchronize to daily and seasonal changes (Harmer et al., 2001; Buhr et al., 2010; Buhr and Takahashi, 2013; Jagannath et al., 2017; Cox and Takahashi, 2019). In the past, the first scientific observations suggesting the presence of a regulatory mechanism inside living beings closely connected to environmental changes were those realized in 1729 (and reported in “*Histoire de I'Academie Royale des Sciences de Paris, Année MDCCXXIX”*) by Jean Jacques d'Ortus De Marian. These scientific observations were accomplished on *Mimosa pudica*, a perennial herbaceous plant belonging to the Mimosaceae family whose leaves showed a capacity to react to tactile stimuli and circadian variations in light intensity ordered by the day/night cycle. Biological clocks might have been present in the earliest life forms and could have endowed pervasively also organisms lacking inner biomembranes, with the aim to separate in the temporal dimension incompatible biochemical reactions or organize chronological sequences of biologically associated processes (photosynthesis and nitrogen fixation, oxidation reduction, and DNA transcription). However, from an evolutionary point of view, endogenous biological clocks could have provided individual and species survival advantages. On the basis of this hypothesis, when the Earth was devoid of an extensive ozone layer, life forms might have emerged only if they could endow an entrainable biological clock driving DNA synthesis nocturnally and in coincidence with a minimal level of ionizing radiation from the Sun. In more recent years, further studies have highlighted the presence of a sort of endogenous peacemaker, a factual biological clock responsible for regulating circadian rhythms in life forms ranging from insects and mammals to plants, fungi, and even in prokaryotic organisms such as cyanobacteria (Iwasaki et al., 2002; Xu et al., 2003; Johnson, 2004; Axmann et al., 2007; Johnson, 2007; Woelfle et al., 2007; Johnson et al., 2008).

### The Circadian Timing System

All physiological and behavioral processes show nycthemeral fluctuations that in mammals, as well as in the subgroup of primates known as the Great Apes and including humans, are managed by a hierarchical organization named circadian timing system (Honma, 2018). This multipart anatomical and cellular architecture comprises a central pacemaker in the ventral hypothalamus operated by the neurons of the suprachiasmatic nuclei (SCNs), entrained by natural or artificial light-related photic stimulus conveyed from the retina via the retino-hypothalamic tract to the SCNs. The central pacemaker located in the SCNs functions as a cellular network capable of generating circadian on/off firing sequences in cell-autonomous and self-sustained mode *in vitro, in vivo,* and *ex vivo*, essential for guiding the rhythmic activity of the whole organism, well beyond the sleep/wake cycle, for instance, daily oscillation of body temperature, serum hormone levels, and metabolic fluxes (Tarquini and Mazzoccoli, 2017). The circadian timing system is commonly described as being constituted by three interrelated elements among which the information flow may be routed upstream and downstream: 1) input pathways, which collect and conduct environmental cues; 2) transducers, which elaborate and integrate the time-related signal (biological clocks); and 3) effector pathways, which convey signals to downstream elements (Ukai and Ueda, 2010). Environmental photic cues are perceived in the inner retina by neurons transferring information from the eye to the brain, the retinal ganglion cells (RGCs). Among these RGCs, some specialized blue-light (lambda_max_ ∼480 nm)-responsive neuronal cells, called intrinsically photosensitive RGCs (ipRGCs), express a visual pigment called melanopsin (encoded by the *Opn4* gene). Light with ∼480 nm wavelength activates melanopsin to trigger a G-protein cascade that causes membrane depolarization (Gooley et al., 2010). These photosensitive ganglion cells project to the SCN directly via the retino-hypothalamic tract. Basically, timekeeping is sustained by the intrinsic activity of autonomous cellular timepieces within the SCN of the hypothalamus, where single SCN neurons tick with robust and synchronized self-sustained oscillations, which allow the SCN to uphold coherent tissue rhythmicity. In sequence, the SCNs drive phase harmonization among cellular self-sustained and cell-autonomous oscillators present in almost every peripheral tissue (Dibner et al., 2010). The SCN drives rest/fasting and activity/feeding cycles and these behavioral rhythms act as the most effective time cue for the biological clocks of body tissues and organs (Kornmann et al., 2007; Bechtold and Loudon, 2013; Mendoza, 2019). Autonomic nervous system fibers of the sympathetic and the parasympathetic limbs, body temperature rhythms, hormones, such as melatonin and cortisol (through glucocorticoid receptors), and the transcription factor serum response factor (SRF) communicate to organ systems phased signals from the SCNs, which on the other hand are not reset/entrained by feeding-related signals (Mendoza, 2007; Challet, 2019; Mistlberger and Antle, 2011; Mohawk et al., 2012; Schibler et al., 2015).

### The Biological Clock

In all the kingdoms of the Tree of Life, convergent evolution drove development of endogenous molecular clockworks, allowing daily and seasonal adjustment of cellular and organismal physiology to environmental cycles. Biological clocks allowed anticipation of rhythmically recurrent ambient changes, conferring advantage over selective pressure and driving natural selection, with emergence of profitable genotypic/phenotypic features (Dunlap, 1999). In almost every mammalian cell and peripheral tissue, rhythmicity of cell processes and physiological functions are driven by molecular clockworks. These peripheral biological oscillators go on ticking even when the SCNs have been surgically ablated in animal models, signifying that within peripheral tissues circadian oscillators are capable to function autonomously. The mechanism operating cellular timekeeping is built predominantly on transcription–translation feedback loops (TTFLs) in which circadian proteins directly or indirectly affect the frequency of expression of the genes that encode them defined as clock genes (Takahashi et al., 2008). Although the proteins encoded from such genes are not conserved across the domains of life and differ among various organisms, their TTFL-related organization and their functions are rather comparable, even if co-evolution of intermingled non-transcriptional (peroxiredoxin proteins) oxidation–reduction cycles have been described as well (Edgar et al., 2012).

### Plants and Molecular Clockworks

Plants are equipped with endogenous biological clocks driving circadian and seasonal oscillations of photosynthesis and several other processes throughout the life cycle, such as development, reproduction, as well as flowering, germination, growth, reproduction, and pollination time. The organization of the molecular clockwork in plants, similarly to insects and animals, comprises complex intertwining TTFLs, whose architecture has been mainly investigated in *Arabidopsis thaliana*, extensively used as a model organism in plant biology, and classified in the taxonomic family of the Brassicaceae in the eudicotyledonous group of angiosperm vascular plants. Considering exclusively the core loop, not mentioning epigenetic, posttranscriptional, and posttranslational modifications, the MYB transcription factors CIRCADIAN CLOCK ASSOCIATED 1 (CCA1) and LATE ELONGATED HYPOCOTYL (LHY) are expressed at dawn and repress the expression of TIMING OF CAB EXPRESSION 1 (TOC1) also known as PSEUDO-RESPONSE REGULATOR 1 (PRR1), while at dusk, CCA1 and LHY levels diminish unleashing the expression of TOC1, which in turn represses CCA1 and LHY transcription together with other PRR family members (PRR9/PRR7/PRR5). Multiple interlocked negative feedback loops are driven by GIGANTEA (GI), EARLY FLOWERING (ELF), and LUX ARRHYTHMO (LUX), as well as REVEILLE (RVE), LIGHT-REGULATED WD (LWD), and NIGHT LIGHT-INDUCIBLE AND CLOCK-REGULATED (LNK) that work as positive regulators (Sanchez and Kay, 2016).

### Insects and Molecular Clockworks

In *Drosophila melanogaster*, the more frequently studied insect model, the transcription factors Clock (Clk) and Cycle (Cyc) set out the expression of period (Per) and timeless (Tim) encoding genes, and successively Per and Tim proteins accrue overnight and suppress Clk/Cyc activity through direct Per-Clk/Cyc interactions and Clk/Cyc phosphorylation. Sunlight affects in a direct way the biological clock in *Drosophila melanogaster* mainly activating cryptochrome (cry), an inner brain blue-light photoreceptor expressed rhythmically with circadian pattern, whose activation by light causes rapid Tim degradation at dawn and retunes daily the molecular clockwork (Emery et al., 1998).

### Mammals and Molecular Clockworks

**FIGURE 1:**
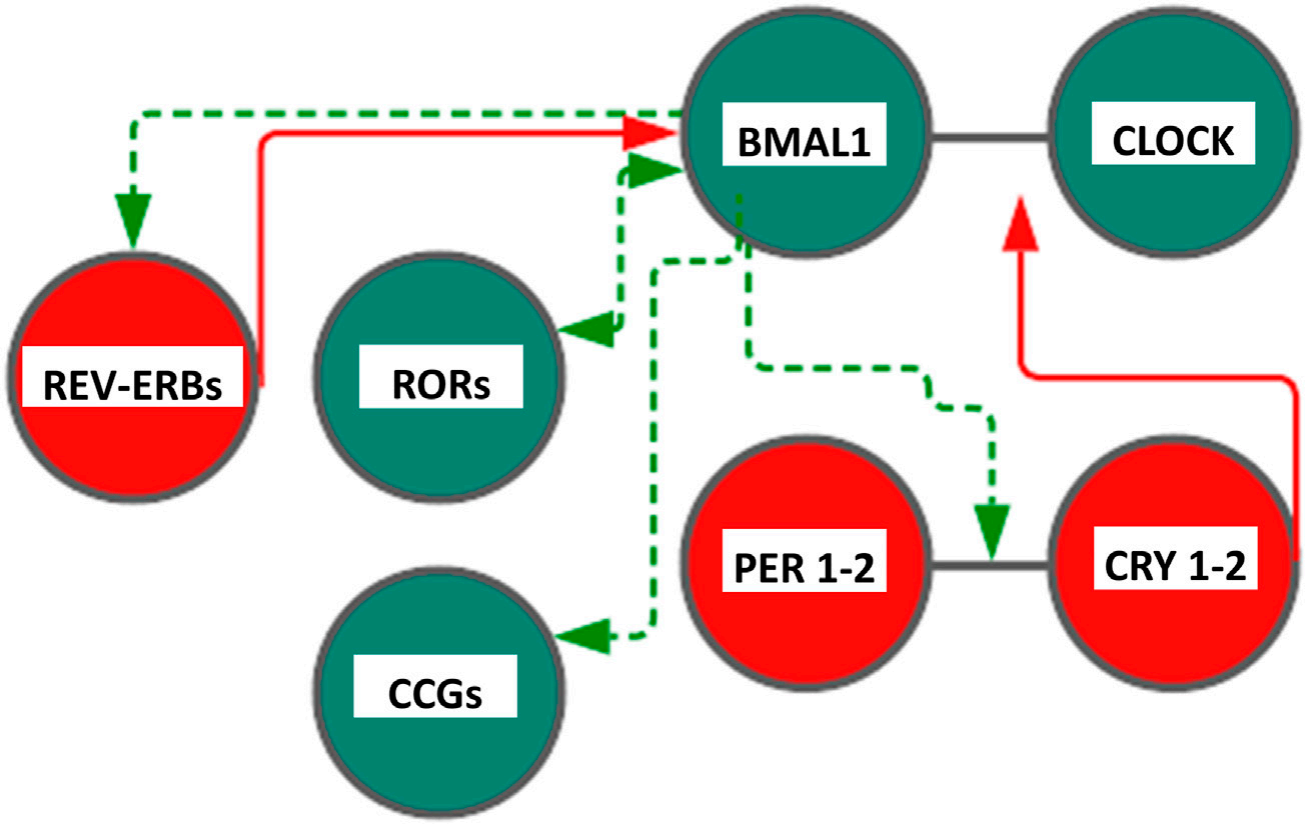
Scheme representing the core cogs of the mammalian molecular clockwork. The transcriptional/translational feedback loop entails a positive (activating) limb operated by BMAL1:CLOCK heterodimer transcriptional activity and a negative limb (inhibitory) operated by the PER:CRY heterodimer. Rhythmic BMAL1 expression is driven by an additional loop through the nuclear receptors REV-ERBs and RORs. Color code: red circles and lines indicate inhibitory activity, and green circles and lines indicate activating function. CLOCK—Circadian Locomotor Output Cycles Kaput), BMAL1—Brain and Muscle Arnt-Like protein 1, PER1-2—(Period proteins), CRY1-2—Cryptochrome proteins , REV-ERBs—Reverse transcript of erythroblastosis geneα, β, and RORs—RAR-related orphan receptor α, β, and γ proteins. See text, for more explanations.

In mammals, similarly, the TTFL is operated by a positive limb and a negative limb managed by the heterodimers CLOCK/BMAL and CRY/PER, respectively, making going on the feedback loop circuits. In the first loop, heterodimerization at the cytoplasmic level of two helix–loop–helix proteins (helix–loop–helix, HLH), Circadian Locomotor Output Cycles Kaput (CLOCK, or the analog NPAS2 in neuronal tissues) and Brain and Muscle Arnt-Like protein 1 (BMAL1), allows their penetration into the nucleus, and the link with the E-box sequences in clock and clock-controlled genes promoters thus allowing the expression of Period proteins (PER1, PER2, and PER3) and Cryptochrome proteins (CRY1 and CRY2) thanks to the rhythmic activation of the respective genes. The repression of this mechanism with consequent closure of the loop occurs when the accumulation of PER and CRY proteins in the cytoplasm leads to the formation of heteropolymeric complexes of unknown stoichiometry that return to the nucleus to inhibit the transactivation of CLOCK:BMAL1 (Gachon et al., 2004; Nagoshi et al., 2004). In addition to this first loop, a second circuit is associated to drive the expression of *BMAL1* gene: CLOCK:BMAL1 heterodimer, in fact, promotes the transcription of REV-ERB (α, β orphan nuclear receptor) and ROR (α and γ) proteins, belonging to the family of orphan nuclear receptors connected to retinoic acid. REV-ERB and ROR, therefore, become an integral part of the loop as important regulation elements (Hardin and Panda, 2013). Specifically, they bind to specific response elements (retinoic acid-related orphan receptor response elements, ROREs) on *BMAL1* promoters allowing, respectively, repression or the activation of transcription of *BMAL1* gene. The general mechanism is very complex and has important repercussions at the level of cellular metabolism. Indeed, CLOCK:BMAL1 activates the rhythmic expression of numerous downstream genes encoding functional proteins managing rhythmic patterns and crucial cellular processes (Duong et al., 2011; Koike et al., 2012; Ye et al., 2014). Posttranslational modifications (PTMs) of clock proteins adjust the molecular clockwork functioning and protein phosphorylation is one of the most important PTMs; for example, serine/threonine protein kinase casein kinase I ε (CKIε) is a key regulator of period length, binds to and phosphorylates BMAL1 and the PERIOD and CRYPTOCHROME proteins, as well as multiple other circadian cogs (Eide et al., 2002; Gallego and Virshup, 2007; Gao et al., 2013). CLOCK:BMAL1 heterodimers drive the circadian transcription of downstream effector genes, known as clock-controlled and tissue-specific output genes, some of which are encoded by the transcription factors DBP, HLF, TEF, DECs, and the REV-ERBs nuclear receptors, implicated in multiple physiologic functions (Yamaguchi et al., 2000). DBP (albumin D-site-binding protein), HLF (hepatic leukemia factor), and TEF (thyrotroph embryonic factor) are the three members of the proline and acidic amino acid-rich basic leucine zipper (PAR bZip) transcription factor family, whose expression is under direct circadian control, with transcription driven by the CLOCK:BMAL1 dimers and suppressed by PER:CRY heterodimers (Falvey et al., 1995; Fonjallaz et al., 1996). E4BP4 protein is closely related to DBP. Its promoter contains a RORE element, making it susceptible to transcriptional suppression by REV-ERB’s. Hence, although E4BP4 expression follows an oscillatory pattern, its phase is opposite to that of DBP. Besides, the basic helix‐loop‐helix transcription factors DEC1 and DEC2 are reported to be circadian components involved in the molecular clockwork. The orphan nuclear hormone receptors REV-ERBα. and REV-ERBβ act as negative transcriptional regulators by binding ROR-specific response elements (RORE) in gene promoters, thus preventing the binding of the positive transcription regulator RORα (Takahashi 2017). An additional level of regulation is represented by SIRT1 (silent mating type information regulation 2 homologue, homolog 1), a type III nicotinamide (NAM) adenine dinucleotide (NAD+)-dependent protein, and histone deacetylase (HDAC), impacting the molecular clockwork functioning at different levels: 1) deacetylating histone H3, which induces cycles of chromatin modifications and remodeling with epigenetic regulation of transcriptional activity; 2) propping up the amplitude of transcription for numerous core clock genes, counting BMAL1, PER2, and CRY1; and 3) deacetylating with circadian pattern BMAL1 and PER2, decreasing PER2 stability. SIRT1 activity hinges on intracellular levels of NAD+, and NAD+/NADH ratio gauges cellular oxidation–reduction and metabolic activity. Cytosolic and nuclear NAD + levels go through circadian fluctuations driven by 24-h periodicity of expression of the rate-limiting enzyme driving the NAD + salvage pathway, nicotinamide phosphoribosyltransferase (NAMPT), encoded by a clock-controlled gene under direct BMAL1 transcriptional control. Interestingly, *in vitro* experiments showed that DNA-binding activity of CLOCK:BMAL1 heterodimers is influenced by NAD(P)+/NAD(P)H ratio. (Asher et al., 2008; Nakahata et al., 2008; Nakahata et al., 2009; Ramsey et al., 2009; Asher and Schibler, 2011; Sahar and Sassone-Corsi, 2013). NAD+/NADH ratio is also adjusted through NAMPT activation by AMP-activated protein kinase (AMPK), serving as an additional nutrient sensor and regulator of biological clock pacing through CRY1 and CRY2 protein phosphorylation, with tagging for degradation (Fulco et al., 2008; Lamia et al., 2009; Jordan and Lamia, 2013) (Figure 1).

## Quantum Biology

**FIGURE 2:**
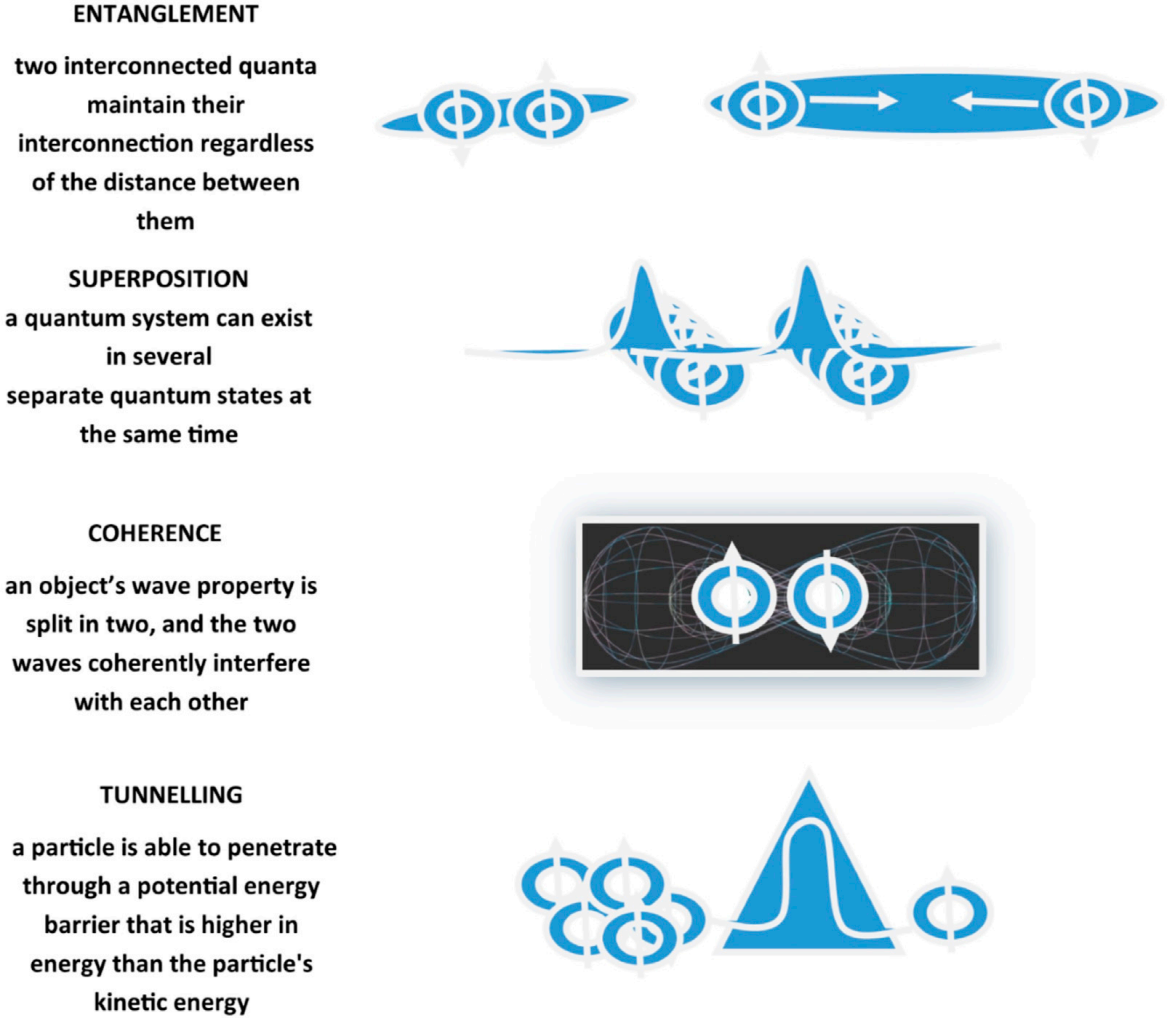
Schematic representation of quantum mechanical effects including entanglement, coherence, superposition, and tunneling. Entanglement, also defined as quantum correlation, is the ability of two quanta interconnected with each other to maintain the interconnection regardless of the distance that separates them. Coherence refers to the ability of a quantum state to maintain its entanglement and superposition despite interactions. Superposition is the ability of a quantum system to be in multiple states at the same time until it is measured. Tunneling is a quantum mechanical phenomenon related to the behavior of a quantum particle at a potential barrier through which a wave function can propagate even though particle total energy is less than the barrier height.

Recurring chemical processes sustain life and make it possible for organisms to stay alive. First and foremost, biology is propelled by chemistry, which covers composition, structure, properties, behavior, and reshuffling of atoms, molecules, and ions upon reaction with other substances, and chemistry obeys the rules of quantum mechanics. The study of applications of quantum mechanics and theoretical chemistry to biological phenomena and processes represents the field of interest and research in Quantum Biology (Longuet-Higgins, 1962; Wolynes, 2009). Quantum Biology scrutinizes non-trivial quantum effects in biological processes exploiting fundamental physics laws, modeling biological systems through computations taking into consideration quantum mechanical effects (Table 1). Effectively, experimental data imply the likelihood, at least at some point in a range of biological processes, of quantum entanglement, quantum superposition, quantum coherence, and quantum tunneling (Ball 2011; Lambert et al., 2013; Marais et al., 2018; Konik, 2021) (Figure 2). The main source of energy that fuels chemical reactions in biosystems and is incorporated and accumulated by living organisms in the form of nutrients is supplied by solar radiation. Transient excited states with chemical and physical features significantly diverging from the original molecules are elicited by absorption of electromagnetic radiation, mainly but not exclusively of the visible light spectrum. Chemical reactions prompted and got going by the absorption of energy in the form of light from the solar flux are defined photochemical reactions, for example, photosynthesis. These biological processes encompass quantum mechanical transformation of energy into forms that are functional for chemical conversions and implicate light absorption, excited electronic states creation, excitation energy conveyance, and electrons/protons relocation in chemical reactions (Ball 2011; Lambert et al., 2013; Marais et al., 2018). Quantum Biology represents an emerging area of research, at present proposing theoretical frameworks reliant on proceeding investigations, but thought on all over the 20th century by forerunner quantum physicists, such as Erwin Schrödinger, Niels Bohr, Pascual Jordan, and Max Delbruck, who envisaged and proposed potential relevance of quantum mechanics in biological phenomena. On the occasion of lectures delivered under the auspices of the Dublin Institute for Advanced Studies at Trinity College, in February 1943 and published in 1944 in the book “*Was ist Leben - What Is Life? The Physical Aspect of the Living Cell*” (Schrödinger, 1944), Schrödinger suggested the concept of genetic information stored in life forms through an arrangement of covalent chemical bonds in DNA base pairs just like in an “aperiodic crystal” in which “quantum leaps” could trigger mutations (Varn and Crutchfield, 2016). Successively, Löwdin proposed proton tunneling as an additional DNA mutation mechanism, foreseeing Quantum Biology as a new field of study (Löwdin, 1963).

**TABLE 1:** Glossary of chronobiological and quantum mechanical terms

Chronobiology	The scientific discipline that studies the time-related features of biological processes
Circadian rhythm	A biological cycle with ∼24-hour time interval
Biological clock	Molecular clockwork hardwired by genes and proteins operating a transcriptional and translational feedback loop with adjustable time delay
Quantum Biology	The study of applications of quantum mechanics and theoretical chemistry to biological phenomena and processes
Entanglement	The link of a fundamental nature existing between constituent particles of a quantum system through which the quantum state of each constituent of the system instantly depends on the state of the other constituents
Superposition	The ability of a quantum system to be in multiple states at the same time until it is measured
Coherence	The ability of a quantum state to maintain its entanglement and superposition in the face of interactions and the effects of thermalization
Tunneling	The ability of a particle to have a determinate possibility of crossing an energy barrier
Photosynthesis	The process occurring in eukaryotic organisms through which light energy is transformed into chemical energy
Frenkel exciton	Electron–hole pair of small radius in a single atomic site
DNA mutation	Alteration of DNA sequence from point mutation and base substitution hindering base pairing
Phototransduction	The photochemical reaction occurring when light energy conveyed through the optical system of the eye is transduced into an electric phenomenon in the retina
Magnetoreception	A type of sensory perception based on sensing by living beings of spatial alignment of the geomagnetic field lines through radical-pair and spin-chemical-related processes
Radical pair	Product of spin-conserving electron transfer reaction in a molecule with an odd number of electrons

## Photosynthesis

Photosynthesis is the process through which light energy is transformed into chemical energy, occurring in eukaryotic organisms of the *Viridiplantae* kingdom, also called *Chlorobionta*, comprising two phyla: *Chlorophyceae* (diatoms and green, yellow–green, red, brown, and golden algae) and *Streptophyceae* (charophyte algae and embryophytic land plants, including liverworts, mosses, hornworts, lycopods, ferns, gymnosperms, and flowering plants)(Simpson, 2019). In photosynthetic organisms, light energy (photons) is absorbed through electronic excitation in various light-harvesting apparatus chromophores, called antennae, and represented, for instance, by multifeatured symmetrical circular structures in purple bacteria, phycobilin in *cyanobacteria,* and chlorophyll pigments in green plants. Overall, photosynthesis generates Frenkel excitons, bound electron–hole pairs held in a chromophore (Lloyd, 2011). Exciton is a bound state of an electron (negatively charged) and a hole (positively charged) which are strongly attracted to each other by the electrostatic Coulomb force between them. It is present in all systems where electrons can be excited to higher states and is an electrically neutral quasi-particle with slightly less energy than the unbound electron–hole state that can transport energy and momentum and move freely through the medium or can be scattered by impurities. Excitons are metastable with a finite lifetime (from picosecond to few nanosecond recombination lifetime) and the excited electron can fall back into the empty hole with emission of a photon. Frenkel excitons are transferred to a reaction center where charge separation permits energy transformation into functional chemical energy in the cells, with electronic quantum coherence allowing fast excitation energy transfer to reaction centers, avoiding conversion into thermal vibration or fluorescence emission. In *cyanobacteria*, photoautotrophic prokaryotes performing oxygenic photosynthesis, the dominant photosystem is represented by Photosystem I (PSI), which in thylakoids assembles in well-organized and far-reaching macromolecular arrays, affording maximal packing efficiency more than energy transfer (MacGregor-Chatwin et al., 2017). Higher photosynthetic organisms contain *chlorophyll a* and *chlorophyll b*, double-membrane-bounded chloroplasts to yield and stockpile photosynthetic products, and cell walls are mainly made of cellulose. The main energy transformation reactions of photosynthesis take place in the chloroplast thylakoids, photosynthetically active, and highly folded membranes with piled (grana) and non-piled (stromal) lamellae sections compactly filled with photosynthetic complexes (Albertsson, 2001). These comprise chlorophyll and membrane protein (photosystem I, cytochrome b6f, photosystem II, and ATP synthase). Photosystem I (PSI) and photosystem II (PSII) gather sunlight energy and employ it to reduce electron acceptors, to yield the NADPH essential to fix CO2. Appropriate pigment–pigment and protein–protein interactions in thylakoids are essential to attain utmost efficiency of photosynthetic reactions. In thylakoid membranes of higher plants, photosystems I and II perform linear electron transport in the grana, while in the stroma lamellae, cyclic electron transport is performed by photosystem I, which endows a greater quantity of pigments and captures a greater amount of quanta with respect to photosystem II (Albertsson, 2001). Sunlight is efficiently kept by photosynthetic complexes and the excitation energy is transported to and deep rooted in reaction centers, where energy is transformed into useful chemical products through charge separation. Spectroscopical analysis at 77 K (–196 °C) showed superpositions of excitonic states as slowly dephasing quantum beats in the two-dimensional electronic spectra of the Fenna–Matthews–Olson (FMO), the trimeric bacteriochlorophyll *a* complex of the thermophilic green sulfur bacterium *Chlorobium tepidum* (inhabiting dimly lit deep oceans), which transfers energy with nearly 100% efficiency from the chlorosome (a photosynthetic light-harvesting antenna complex) to the reaction center (the site of the light reactions of photosynthesis). The study suggests that energy conveyance is seemingly operated by quantum mechanics models: energy transfer does not happen through a classical random walk-like transmission of the exciton to the reaction center, but surprising evidence of coherent quantum energy transfer was shown (Engel et al., 2007; Ishizaki and Fleming, 2009). Excitations move by multiple trails simultaneously to get to the reaction center and wavelike characteristics upkeep the striking efficiency of this energy transfer process, profiting the phenomenon of long-term quantum coherence and electronic quantum states progressively evolving superpositions, which allow to exploit countless energy-transmitting pathways at the same time. (Engel et al., 2007; Panitchayangkoon et al., 2010). However, excessively rapid decay on a timescale of 60 femtoseconds was recently reported, jostling the hypothesis of long-term quantum features supporting electron transfer to the reaction center (Duan et al., 2017), even if sufficiently long-lasting coherences were recently shown in chlorophyll complexes (Thyrhaug et al., 2018).

### Photosynthesis and Circadian Rhythms

At present, the essential role of quantum effects in photosynthesis, at least during energy transfer after light harvesting, represents a topic of active debate, with the assessment of different representations, i.e., electron tunneling, environmental noise pushing electron quantum walks, geometric symmetries in the complex allowing quantum networks-like perfect state transfer, vibrational motion, and nuclear dynamics in chromophores. In higher plants, photosynthesis shows patterns of circadian rhythmicity driven by the molecular clockwork, which controls rhythmic 24-h oscillation in the quantity of photosynthesis proteins localized in the chloroplast. Approximately 70% of chloroplast transcripts show circadian regulation and a nuclear-encoded regulator of chloroplast genes transcription, SIGMA FACTOR5 (SIG5), functioning as a circadian signal, joins the biological oscillator with chloroplasts allowing adjustment of photosynthetic efficiency according to diel and temporary fluctuations of environmental light intensity (Noordally et al., 2013). In turn, carbohydrates levels signal back to the molecular oscillator gauging photosynthesis activity and chloroplast glucose production (Belbin et al., 2017). Besides, photosynthetic electron transport generates backward signaling that regulates the alternative splicing of nuclear-encoded transcripts encoding splicing factors, such as SR protein among others, triggered by depletion of plastoquinones pool (Petrillo et al., 2014; Dodd et al., 2015).

## DNA Mutation

The material containing hereditary information transmitted from one generation to the next is constituted, in all living organisms, of a nucleic acid called deoxyribonucleic acid (DNA). Genetic information is stored in DNA strands according to a code based on four nitrogenous bases, called adenine (A), thymine (T), guanine (G), and cytosine (C), which forms a nucleotide when linked with deoxyribose and a phosphate group allowing nucleotides to unite among them by phosphodiester covalent bonds. Every DNA molecule is double stranded, i.e., two complementary helices of nucleotides are coupled by hydrogen bonds between G-C and A-T base pairs, with entanglement suggested to play a key role in establishing the stability of the DNA double helix (Rieper et al., 2010).

Alteration of DNA sequence, defined as point mutation, can be caused by DNA copying faults through cell division, chemical damage (mutagens, carcinogens), virus infections, ionizing radiation (gamma rays and X-rays), or ultraviolet (UV) light (UVA 315–400 nm, UVB-280–315 nm, and UVC 100–280 nm), with cyclobutane pyrimidine dimer (CPD) or pyrimidine (6,4)–pyrimidone (6-4 PP) as the main DNA photochemical product (Pfeifer et al., 2005). A model of DNA mutation, through which base substitution thwarts base pairing with universal G:C → A:T mutation bias in all life kingdoms and modification of the DNA strand structure, invokes interbase double proton transfers (DPT) and quantum-mechanical tunneling effect with impact on genetic stability (Löwdin, 1963).

Proton tunneling in DNA involves tautomeric forms of bases which are not easy to determine experimentally and the use of computational tools is often involved. The tools of quantum information theory have made it possible to highlight that bi-directional quantum tunneling events take place between chemically bonded atoms, but a useful quantum entanglement cannot be generated due to the oneness of electrons in case of tunneling of an electron pair defining a covalent bond. However, an effective quantum entanglement can be acquired by a covalent bond if it has a particular quantity of ionic character, especially if it involves an electronegative atom and a classic H bond. On the other hand, in addition to thermal equilibrium, an effective quantum entanglement can be generated by tunneling the electrons of the proton acceptor atom or the proton of the H atom into a partially covalent H bond. Besides, a decrease in the amount of ionic character of the proton donor covalent bond increases the amount of this entanglement. The effective entanglement shared in partially covalent H bonds could be used in biosystems, particularly in the molecular recognition processes involved in DNA replication (Pusuluk et al., 2018).

Experiments performed in *Escherichia (E.) Coli* grown in deuterium oxide-enriched media showed decreased spontaneous mutation rate; considering that quantum tunneling probability is inversely correlated with the mass of the particle, replacement of the normal hydrogen atom from the DNA with its heavier isotope deuterium reduces the probability of this quantum phenomenon, so that the results observed in *E. Coli* cultured in deuterated environment suggest a role played by diminution of quantum tunneling (Srivastava, 2019).

### DNA Mutation and Circadian Rhythms

Altered base pair bonds are re-established in a (blue) light-dependent manner by photolyases, DNA repair enzymes dependent on the cofactor flavin adenine dinucleotide (FADH), through a cyclic electron transfer process called photoreactivation, which splits thymine dimer bonds and re-establishes DNA integrity. Photolyase is excited by blue light and transfers an electron to the cofactor FADH-, which then donates the electron through quantum tunneling to the photoproducts dimer to break the bond and repair DNA (Thompson and Sancar, 2002). Photolyases and cryptochromes belong to the photolyase/cryptochrome family of flavoproteins, which bind two chromophores, flavin adenine dinucleotide (FAD) and 5,10-methenyltetrahydrofolate (MTHF) or 8-hydroxy-7,8-didemethyl5-deazariboflavin, with their well-conserved ∼500 amino acids core domain. A molecular clockwork regulatory function of photolyases, precisely with repressor role, has been described for the diatom *Phaeodactylum tricornutum*, the green alga *Ostreococcus tauri*, the rat-kangaroo *Potoruos tridactylus,* and a nucleopolyhedrovirus (ChchNPV) infecting the lepidopterans *Chrysodeixis chalcites*, This baculovirus expresses the photolyase-like genes *phr1* and *phr2*, encoding CPD photolyase PHR1 and 6-4 PP photolyase PHR2, the latter showing ability to bind the CLOCK protein and to impede *in vitro* CLOCK/BMAL1-driven transcription in HEK 293T cells and NIH 3T3 cells (Biernal et al., 2012). Interestingly, experiments performed in human quiescent fibroblasts, synchronized with a dexamethasone pulse, showed that the severity of UV-light induced DNA damage and the proficiency of the Nucleotide Excision Repair process depend on circadian time of exposure and time-related chromatin remodeling, mainly through relaxation induced by histone acetylation, both driven by a functioning biological clock (Kang and Sancar, 2009; Bee et al., 2015; Kang, 2021). The Nucleotide Excision Repair process works through the nanomachinery correcting UV-light induced DNA photolesions (along with DNA bulky adducts from chemicals, base alterations form reactive oxygen species and intrastrand DNA crosslinks from various exogenous or endogenous agents) (Kang and Sancar, 2009; Bee et al., 2015; Kang, 2021).

## Visual Phototransduction

Phototransduction is the photochemical reaction occurring when light energy conveyed through the optical system of the eye is transduced into an electric phenomenon in the retina starting with an electrochemical signal sent by way of the ganglion cells to the brain through the optic nerve. In the absence of light, the photoreceptors are depolarized to a membrane resting potential of −40 mV, while light hyperpolarizes the plasma membrane of the photoreceptor to −70 mV. Following light absorbance, a photon is captured by a photoreceptor (Sia et al., 2014). Opsin molecules, membrane proteins located in the outer segments of the photoreceptors, contain a light-absorbing chromophore (11-cis retinal, an aldehyde of vitamin A) covalently bound to opsin. The photon interacts with 11-*cis* retinal, which undergoes photoisomerization to all-*trans* retinal and induces structural change in the photoreceptor, activation of G-protein-coupled receptors (GPCRs) and cyclic guanosine 3′-5′ monophosphate (cGMP)-related signal transduction pathways, and arising of a visual signal (Sia et al., 2014). Photoisomerization of 11-*cis* retinal to all-*trans* retinal occurs with energy conversion from a resting ground state to a higher energy excited state, indicating non-trivial quantum effects. The process takes place in less than 200 femtoseconds and with high specificity and invariant *ϕ*(λ) (wavelength-dependent efficiency) for blue light exposure (*ϕ* = 0.65 ± 0.01 between 450 and 500 nm), with quantum yield reducing by up to 5% at higher wavelengths (between 500 and 750 nm). Moreover, energy transfer in phototransduction triggers rhodopsin conformational change and successively signal transduction, possibly involving conformational quantum superposition and quantum coherence effects (Sia et al., 2014).

### Visual Phototransduction and Circadian Rhythms

Retinal physiology shows circadian patterns of variation. Efforts to recognize the retinal cells capable to drive rhythmicity have produced contradictory results, despite that retinal cell types generally express core clock genes and, in the retina, are present all the components of a circadian clock circuitry, specifically neuroepithelial cells accomplished to oscillatory phototransduction (Ruan et al., 2006; Tosini et al., 2007; Ruan et al., 2008). Among the candidate circadian factors, photoactive cryptochrome proteins play different and independent roles in driving 24-h rhythmicity in retinal physiology, mainly related to CRY1, while CRY2 seems in a certain way less essential. Studies performed in mouse retina showed that CRY1 is expressed in cone photoreceptors, amacrine cells, and retinal ganglion cells and its deletion dampens or impedes pupillary light responses and retinal rhythms, such as contrast sensitivity rhythm and photopic electroretinogram b-wave amplitude. On its side, CRY2 retinal cell localization was limited to the photoreceptor layer and its deletion attenuated only photopic electroretinogram b-wave amplitude (Wong et al., 2018).

## Magnetoreception

**FIGURE 3:**
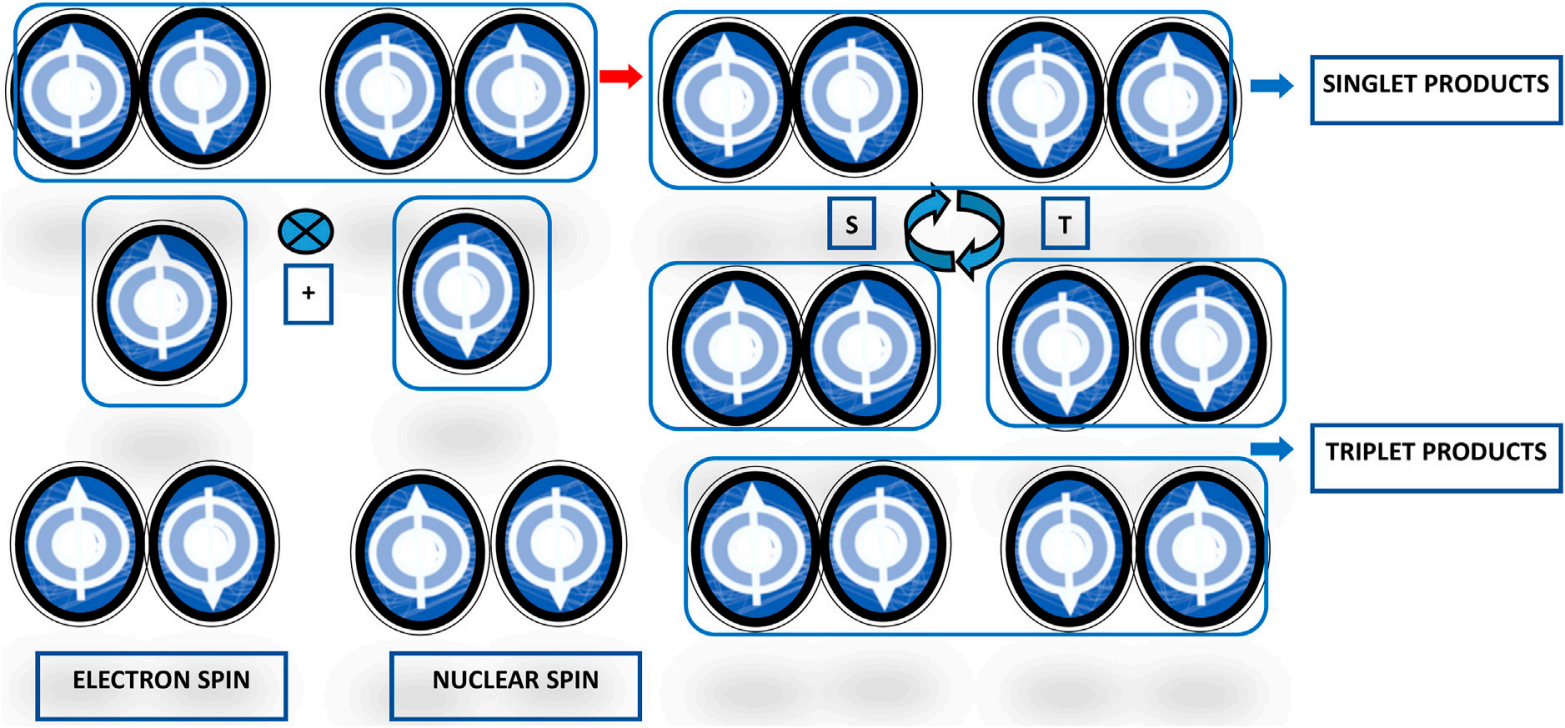
Scheme representing the radical-pair reaction mechanism for avian magnetoreception. The basic reaction scheme of the magnetoreception in birds encompasses light absorption by and chemical reaction of the radical pair in Cryptochrome molecules in the eyes. Activation by photons with adequate energy elicits an electron transfer reaction producing the radical pairs in their excited singlet states. The state of the pair can remain in a singlet state or become a triplet state, with different singlet- and triplet-state ratios depending on the geomagnetic field influence.

Magnetoreception is a type of sensory perception based on sensing by living beings of spatial alignment of the geomagnetic field lines (for an accurate description of the Earth’s magnetic field refer to https://web.ua.es/docivis/magnet/earths_magnetic_field2.html). In migrating and non-migrating animals, magnetoreception pivots on sensitivity to axial course and inclination in space of geomagnetic field lines. Examples of animal magnetoreception are found in all major branches of the animal kingdom, including really different species ranging from migratory birds to fishes, crustaceans, amphibians, and insects, such as robins, silvereyes, garden warblers, salmons, spiny lobsters, sea turtles, spotted newts, honeybees, beetles, cockroaches, fruit flies, dragonflies, butterflies, and moths, among others. Animal species use geomagnetic field sensing for orientation and navigation by way of the axis and dip angle of the Earth’s magnetic field lines, using the eyes as the site of magnetoreception, seemingly through the radical pair and spin-chemical processes in cryptochromes and chromophores as phototransducers, involving enduring quantum phenomena such as quantum coherent superposition and entanglement (Cai et al., 2010; Gauger et al., 2011). This basic mechanism works as an inclination only compass through electron spin-dependent photochemical compounds production in response to geomagnetic field lines axis and dip angle. Photon absorption results in the production within the cryptochrome proteins of a couple of radicals, molecules with an unpaired electron each, possibly entangled, charging a radical pair with two possible states, singlet (*S0*) with antiparallel spin and triplet (*T0*) with parallel spin. The radical pair oscillates between these two states (just like Bell states and quantum bits), with the singlet-to-triplet ratio depending on the radical-pair orientation with respect to the geomagnetic field lines direction (Figure 3). Birds perceive the Earth’s magnetic field exploiting photochemical transformations in cryptochrome flavoproteins. Blue or green light absorption produces a radical pair of electrons in an oriented cryptochrome molecule within the bird’s retina. The radical pair goes through coherent quantum wavering between entangled singlet and triplet states and a spin-dependent electron transition then allows current to flow only if the spins in the pair are in a particular symmetry state. The cryptochrome molecule orientation with respect to the Earth’s magnetic field determines the rate of current flow (Lloyd, 2011). The radical-pair singlet and triplet states join with different biochemical reactions, whose products modulate neural signaling from the avian retina to the brain and render information about the geomagnetic field lines direction (Ritz et al., 2004; Ritz et al., 2009; Guerra et al., 2014; Wiltschko and Wiltschko, 2014; Wiltschko et al., 2021).

### Magnetoreception and Circadian Rhythms

The avian magnetic compass has been deeply investigated and is hallmarked by light dependence and insensitivity to polarity. Magnetoreception in orientation behavior is generally not possible in the dark (even if many land birds migrate at night), light from the short-wavelength end of the spectrum is needed and the direction of very weak magnetic fields may affect radical-pair electrons reactivity and, consequently, the way they catalyze biochemical reactions/products (Wiltschko and Wiltschko, 2014; Wiltschko et al., 2021). The interaction of entangled electron pairs with the Earth’s magnetic field changes the ratio between singlet and triplet pairs and, in turn, the alteration of the singlet–triplet swapping dynamics changes the production of the reaction products, with response degree hinging on the orientation of the radical pair with respect to the direction of the geomagnetic field lines products (Wiltschko and Wiltschko, 2014; Wiltschko et al., 2021). Magnetically sensitive radical pairs are generated in cryptochrome proteins, which are the putative receptor molecules susceptible to being activated by the wavelengths of light that allow magnetic compass orientation, with cryptochrome-dependent magnetosensitivity not necessarily relying on a properly ticking molecular clockwork, as shown in *Drosophila melanogaster* (Gegear et al., 2008). In eukaryotic organisms, cryptochromes are solely capable to yield radical pairs through photoreduction and oxidation reaction upon interaction with full-spectrum light (white) or a single optical frequency with wavelength up to but not exceeding ∼565 nm, comprising ultraviolet, blue, turquoise, and green light (only temporarily and after exposure to white light in the case of the latter wavelength). Flavin adenine dinucleotide (FAD), the chromophore of cryptochrome protein, goes through a redox cycle, with a recurring sequence of photoreduction and re-oxidation (producing the FADox, the dark resting form, and in sequence FAD (•) (-) and FADH (-) redox forms of flavin), and during this photocycle radical pairs are produced (Wiltschko and Wiltschko, 2014; Wiltschko et al., 2021). A light-induced electron-transfer reaction produces a spin-correlated radical pair with singlet and triplet states and this cryptochrome-based radical-pair sensor transduces the effects of the geomagnetic field on the photochemistry of the radical pair (Wiltschko and Wiltschko, 2014; Wan et al., 2021; Wiltschko et al., 2021). The radical pair must be in a coherent superposition of the quantum states of the two-electron spins and when the spin coherence perseveres more than a few microseconds, the yield of the sensor holds a precise characteristic defined spike. When the quantum mechanical spin energy levels of radicals formed in cryptochromes escape crossings, then the spike is activated, potentially conveying a directionality accuracy suitable to clarify the navigational behavior of migratory birds in the wild (Hiscock et al., 2016). In the retina of birds, five isoforms of cryptochromes are found: cryptochrome 1a (Cry1a), cryptochrome 1b, cryptochrome 2, cryptochrome 4a, and cryptochrome 4b. The currently reliable scientific data points to Cry1 or Cry4 as the likely receptors for sensing directions. Cry1 is expressed with circadian rhythmicity and could mediate in a time-related manner the effects of the geomagnetic field on the photochemistry of the radical pair (Wiltschko and Wiltschko, 2014; Wiltschko et al., 2021). However, actually Cry4 is becoming a stronger magnetoreceptor candidate than Cry1 isoforms, as independent groups were not able to purify Cry1 isoforms with FAD content, which puts a question mark on the nature of Cry1s in being a real photosensor (Ozturk, 2021; Warrant, 2021). On the other hand, another hypothesis suggests that the avian light-induced biochemical radical-pair mechanism for magnetoreception should rely on constantly expressed retinal cryptochromes. The expression of Cry1, Cry2, and Cry4 genes was measured over a 24-h daily cycle in the retina of the migratory bird *Taeniopygia guttata* (zebra finch) and Cry1 and Cry2 showed circadian oscillation, whereas Cry4 expression was not rhythmic, suggesting it could be the best possible magnetoreceptor for light-dependent magnetic compass in birds, according to this alternative hypothesis (Pinzon-Rodriguez et al., 2018). Likewise, studies performed in *Erithacus rubecula*, the European robin, migrating during the night, revealed *in vitro* magnetic field sensing operated by Cry4, with a crucial role played by a photoreduction pathway, routed by flavin–tryptophan radical pairs in a four-tryptophan chain, in producing magnetic field influences as well as in balancing and facilitating detection of potential signals (Xu et al., 2021). At this time, a magnetic sense is not supposed to be present in human beings; however, evidence that human CRY2, which shows abundant retinal expression, can work as a magnetosensor in a light-dependent manner in the magnetoreception system was revealed in experiments using transgenic *D. melanogaster*-expressing human CRY2 (Foley et al., 2011). Biological effects in humans might be triggered by lengthy exposure to weak (∼1 μT) to extremely low-frequency (ELF, 50/60 Hz) magnetic field through biophysical mechanisms implicating radical pairs as short-lived chemical reaction intermediates and compatible with the effects of a static magnetic field of the same strength as the geomagnetic field (∼50 μT) (Hore, 2019). In previous studies, solar cycle-dependent geomagnetic field disturbances were correlated with retrospectively assessed long-term variability of amount and hematopoietic cell content of umbilical cord blood (UCB), derived from approximately 18,000 UCB donations collected in Cord Blood Banks in Northern and Southern Italy from 1999 to 2011 (Mazzoccoli et al., 2016; Scholkmann et al., 2016). The geomagnetic field disturbances for solar cycles were quantified by the *Dcx* index, the corrected and extended version of the disturbance storm time (*Dst*) index, gauging the average deviation in the geomagnetic horizontal component (*H*) from its normal value, obtained as daily values by the *Dcx* index server of the University of Oulu, Finland (http://dcx.oulu.fi). UCB yield fluctuations were associated with distinctive correlation patterns and on circadecadal, yearly, monthly, and diurnal timescales with time-related trends of geomagnetic activity magnitude changes, as modulated by solar activity (Mazzoccoli et al., 2016; Scholkmann et al., 2016). The time-qualified patterns of correlation between UCB yield and geomagnetic activity variations are enticing and the biophysical mechanisms responsible for this relationship need to be further addressed to define biophysical intermediates and explore in detail if and how the flavoprotein cryptochrome could be involved with radical-pair/spin-correlated photochemistry changes and geomagnetic field sensing in humans as well.

## Mitochondrial Respiration

Mitochondria are subcellular organelles that produce the greater part of energy in the cell in the form of chemical ATP with really high (60–70%) efficiency. This efficiency rate is due to a quantum mechanism in the process of intracellular energy conversion, which is actuated by quantum tunneling of electrons and hydrogen ions (H+) through potential barriers. In order to transfer charged particles (electrons, protons, and H+) to the electron transport chain proteins, the mitochondrial inner membrane must be overstepped (Mailloux et al., 2013). Long-range electron tunneling on the order of 15–30 Å plays a role in redox reactions in enzymes of cellular respiration. Oxidation of hydrogen ions from carbohydrates and fats yields mitochondrial ATP through the electron transport chain, in which four protein complexes (named complex I–IV) are entailed in transferring electrons from NADH and FADH2 to molecular oxygen. Electrons from NADH are transferred to NADH dehydrogenase (complex I protein), a big L-shaped inner mitochondrial membrane-bound enzyme (Mailloux et al., 2013). Complex I is crucial in the respiratory electron transport chain by coupling the transfer of electrons from NADH to ubiquinone to produce the proton gradient across the membrane necessary for ATP synthesis. This complex induces two cysteine ligands involved in electron tunneling pathways between neighboring Fe/S clusters, which endow specific electronic properties due to the antiferromagnetically coupled high-spin iron atoms (Hayashi and Stuchebrukhov, 2010). From complex I, electrons are transferred to coenzyme Q to produce CoQH2 and then electrons flow to cytochrome-containing complex III, and next to complex IV, where electrons reduce molecular O2 to an oxygen atom that can bind to the hydrogen ions to produce H2O. Electrons movement through the electron transport chain produces energy and induces proton movement (H+ pumping) out of the mitochondrial matrix into the intermembrane space and this charge movement generates a magnetic field and a capacitance potential energy across the matrix (Mailloux et al., 2013). This pushes ATP synthesis through complex V (ATP synthase), adding one phosphate group to interconvert ADP to ATP and driving protons (H+) back into the matrix in a process identified as oxidative phosphorylation. Lastly, energy signals are transformed into electrochemical outputs for ATP transfer at the outer mitochondrial membrane, which hosts a large channel permeable to anions, cations, and ATP, named mitochondrial porin, a voltage-dependent anion channel (Mailloux et al., 2013).

### Mitochondrial Respiration and Circadian Rhythms

Enzymes involved in main mitochondrial pathways, such as pyruvate metabolism, fatty acids uptake, and oxidation, as well as in the Krebs cycle and the respiratory chain complexes, show variations featured by circadian rhythmicity and Ca2+-mediated signaling plays a role in setting the fluctuation of mitochondrial metabolism well timed with its retroactive tuning of the molecular clockwork (Neufeld-Cohen et al., 2016; Scrima et al., 2020). Furthermore, mitochondrial respiratory activity shows time-qualified oscillations dependent on a functioning molecular clockwork, related to fluctuations in cellular NAD content and BMAL1-driven rhythms of NAMPT and Sirtuins 1/3 expression, with reversible acetylation in the electron transport chain of a single subunit of Complex I (Peek et al., 2013; Cela et al., 2016; Scrima et al., 2016).

## Enzymatic Activity

In complex biological systems, interactions among molecules occur with specialness and rapidness, as in the case of the interactions among enzymes and substrates, envisaged as activation energy (barrier) lowering and reaction rate acceleration accomplished by enzymes through time-honored preferential binding enzyme–substrate interaction at the activated complex, usually known as increased transition-state binding. Enzymes are natural catalytic proteins with active sites held in their inner core that is linked to the encompassing solvent by conduits called tunnels, which assist dynamic shuttling of substrates and products. In addition to this function, they support substrate specificity and enantioselectivity, controlling the access of ligands to the active site while hindering exposure to the solvent-embracing protein surface. In biological catalysis, hydrogen bonds between an enzyme and its substrate trigger tautomerization of the substrate to proceed to the ensuant catalysis, with proton tunneling playing a role in this process (Klinmann and Kohen, 2013). Besides, transition from the minor tautomeric to the major tautomeric form involves quantum system dynamics in driving forward the reaction without demanding any extra catalytic activity, with transition states moving through quantum superposition (Pusuluk et al., 2018). The role of proton tunneling in enzyme catalysis has been examined, especially pinpointing the class of oxidoreductases, which oxidize a substrate by transferring its hydrogen (H) to an acceptor molecule through intermolecular H-bonds. These enzymes endow sizeable separations among redox sites, however, electrons are able to transfer in an exponentially distance-dependent and faintly temperature-dependent manner. Long-distance electrons transfer in oxidoreductases possibly exploits single-step quantum mechanical tunneling. The relationship between protein dynamics and the quantum mechanical hydrogen tunneling hints at the inference of protein dynamics for optimal catalysis attainment. Data-based evidence corroborates ranked thermodynamically equilibrated motions driving H-donor/acceptor distance and active-site electrostatics, establishing a miscellanea of conformations suited for H-tunneling (Klinman and Kohen, 2013). However, H-tunneling reactions are neither universally nor exclusively present in enzyme systems, and in addition to proton-coupled electron transfer, other quantum mechanical–molecular mechanical catalytic mechanisms should be considered as well. Along with quantum effects in the transfer of hydrogen-like species in enzyme-catalyzed reactions, quantum mechanical phenomena driven by protein dynamics can play a pivotal role in enzyme action other than H-transfers and electron transfers in proteins.

### Enzymatic Activity and Circadian Rhythms

Regarding thermodynamic stability and free-energy sceneries, non-rhythmically expressed proteins and proteins expressed with circadian rhythmicity and ultradian pattern (oscillating with a frequency two or three times faster than 1 cycle every 24-h) were analyzed for electrochemical properties and components of Gibbs free energy, calculated in the force field/protein design FoldX 4.0 algorithm (Buß et al., 2018). They showed antithetical patterns of correlation with the free energy value related to interresidue close contacts and represented by electrostatic contribution of interactions at interfaces (interresidue Van der Waals’ clashes and electrostatic interactions). Overall, the results suggested higher thermodynamic stability of proteins oscillating with circadian and ultradian rhythmicity, which include the greater part of catalytic proteins, whose expression is broadly driven by the biological clock (Genov et al., 2019). Sustained quantum entanglement and coherence could have been evolutionarily actuated through tertiary and quaternary structure development of these proteins, which could augment the percentage of the reaction that occurs through hydrogen tunneling. In this regard, proteins encoded by genes with expression featured by 24-h frequency harmonics, and particularly those with peak frequency at 1 cycle per 12-h, are more phylogenetically conserved and yet evolutionarily driven by fitting to rhythmic environmental as well as geophysical changes, primarily light/temperature cycles related to dawn/dusk transition, and could render a peculiar genomic signature of evolutionistic eras (Castellana et al., 2018; Genov et al., 2019).

## Conclusion

Time-related variations at multiomics level rhythmically modify amount and subcellular distribution of structural and functional cellular components providing the molecular scaffold within which electrochemical processes as well as non-trivial quantum phenomena can take place. The latter can occur independently of environmental factors or are induced by the interaction of cellular elements with external impulses of various kinds, more often and more importantly linked to electromagnetic radiation with a specific wavelength range. Quantum features including entanglement, superposition, coherence, and tunneling have been principally advocated in light-harvesting processes, such as photosynthesis and photoreception, as well as in processes related to electromagnetic radiation in the spectrum of visible light and ultraviolet rays, such as magnetoreception and DNA mutation, or not strictly related to exposure to light, such as mitochondrial respiration and enzymatic activity. Chronobiology studies biological rhythms and Quantum Biology studies quantum effects in biological systems. Intracellular space–time fluctuations of biomolecules hosting quantum effects suggest valuable and gainful intermingling between these two scientific disciplines, based on physical science and life science, to conciliate complexity of biosystems and counterintuitivity of quantum mechanics.

## References

[B1] Albertsson P.-Å. (2001). A Quantitative Model of the Domain Structure of the Photosynthetic Membrane. Trends Plant Sci. 6 (8), 349–354. 10.1016/s1360-1385(01)02021-0 11495787

[B2] Asher G. Gatfield D. Stratmann M. Reinke H. Dibner C. Kreppel F. (2008). SIRT1 Regulates Circadian Clock Gene Expression through PER2 Deacetylation. Cell 134 (2), 317–328. 10.1016/j.cell.2008.06.050 18662546

[B3] Asher G. Schibler U. (2011). Crosstalk between Components of Circadian and Metabolic Cycles in Mammals. Cell metab. 13 (2), 125–137. 10.1016/j.cmet.2011.01.006 21284980

[B4] Axmann I. M. Legewie S. Herzel H. (2007). A Minimal Circadian Clock Model. Genome Inf. 18, 54–64. 10.1142/9781860949920_0006 18546474

[B5] Ball P. (2011). Physics of Life: The Dawn of Quantum Biology. Nature 474 (7351), 272–274. 10.1038/474272a 21677723

[B6] Bechtold D. A. Loudon A. S. I. (2013). Hypothalamic Clocks and Rhythms in Feeding Behaviour. Trends Neurosci. 36 (2), 74–82. 10.1016/j.tins.2012.12.007 23333345

[B7] Bee L. Marini S. Pontarin G. Ferraro P. Costa R. Albrecht U. (2015). Nucleotide Excision Repair Efficiency in Quiescent Human Fibroblasts Is Modulated by Circadian Clock. Nucleic acids Res. 43 (4), 2126–2137. 10.1093/nar/gkv081 25662220 PMC4344517

[B8] Belbin F. E. Noordally Z. B. Wetherill S. J. Atkins K. A. Franklin K. A. Dodd A. N. (2017). Integration of Light and Circadian Signals that Regulate Chloroplast Transcription by a Nuclear‐encoded Sigma Factor. New Phytol. 213 (2), 727–738. 10.1111/nph.14176 27716936 PMC5215360

[B9] Biernat M. A. Eker A. P. M. van Oers M. M. Vlak J. M. van der Horst G. T. J. Chaves I. (2012). A Baculovirus Photolyase with DNA Repair Activity and Circadian Clock Regulatory Function. J. Biol. Rhythms 27 (1), 3–11. 10.1177/0748730411429665 22306969

[B10] Buhr E. D. Takahashi J. S. (2013). Molecular Components of the Mammalian Circadian Clock. Handb. Exp. Pharmacol. 2013(217), 3–27. 10.1007/978-3-642-25950-0_1 PMC3762864 23604473

[B11] Buhr E. D. Yoo S.-H. Takahashi J. S. (2010). Temperature as a Universal Resetting Cue for Mammalian Circadian Oscillators. Science 330 (6002), 379–385. 10.1126/science.1195262 20947768 PMC3625727

[B12] Buß O. Rudat J. Ochsenreither K. (2018). FoldX as Protein Engineering Tool: Better Than Random Based Approaches? Comput. Struct. Biotechnol. J. 16, 25–33. 10.1016/j.csbj.2018.01.002 30275935 PMC6158775

[B13] Cai J. Guerreschi G. G. Briegel H. J. (2010). Quantum Control and Entanglement in a Chemical Compass. Phys. Rev. Lett. 104 (22), 220502. 10.1103/PhysRevLett.104.220502 20867156

[B14] Castellana S. Mazza T. Capocefalo D. Genov N. Biagini T. Fusilli C. (2018). Systematic Analysis of Mouse Genome Reveals Distinct Evolutionary and Functional Properties Among Circadian and Ultradian Genes. Front. Physiol. 9, 1178. 10.3389/fphys.2018.01178 30190679 PMC6115496

[B15] Cela O. Scrima R. Pazienza V. Merla G. Benegiamo G. Augello B. (2016). Clock Genes-dependent Acetylation of Complex I Sets Rhythmic Activity of Mitochondrial OxPhos. Biochimica Biophysica Acta (BBA) - Mol. Cell Res. 1863 (4), 596–606. 10.1016/j.bbamcr.2015.12.018 26732296

[B16] Challet E. (2019). The Circadian Regulation of Food Intake. Nat. Rev. Endocrinol. 15 (7), 393–405. 10.1038/s41574-019-0210-x 31073218

[B17] Cox K. H. Takahashi J. S. (2019). Circadian Clock Genes and the Transcriptional Architecture of the Clock Mechanism. J. Mol. Endocrinol. 63 (4), R93–R102. 10.1530/JME-19-0153 31557726 PMC6872945

[B18] Cox K. H. Takahashi J. S. (2021). Introduction to the Clock System. Adv. Exp. Med. Biol. 1344, 3–20. 10.1007/978-3-030-81147-1_1 34773223

[B19] Dibner C. Schibler U. Albrecht U. (2010). The Mammalian Circadian Timing System: Organization and Coordination of Central and Peripheral Clocks. Annu. Rev. Physiol. 72, 517–549. 10.1146/annurev-physiol-021909-135821 20148687

[B20] Dodd A. N. Belbin F. E. Frank A. Webb A. A. R. (2015). Interactions between Circadian Clocks and Photosynthesis for the Temporal and Spatial Coordination of Metabolism. Front. Plant Sci. 6, 245. 10.3389/fpls.2015.00245 25914715 PMC4391236

[B21] Duan H.-G. Prokhorenko V. I. Cogdell R. J. Ashraf K. Stevens A. L. Thorwart M. (2017). Nature Does Not Rely on Long-Lived Electronic Quantum Coherence for Photosynthetic Energy Transfer. Proc. Natl. Acad. Sci. U.S.A. 114 (32), 8493–8498. 10.1073/pnas.1702261114 28743751 PMC5559008

[B22] Dunlap J. C. (1999). Molecular Bases for Circadian Clocks. Cell 96 (2), 271–290. 10.1016/s0092-8674(00)80566-8 9988221

[B23] Duong H. A. Robles M. S. Knutti D. Weitz C. J. (2011). A Molecular Mechanism for Circadian Clock Negative Feedback. Science 332 (6036), 1436–1439. 10.1126/science.1196766 21680841 PMC3859310

[B24] Edgar R. S. Green E. W. Zhao Y. van Ooijen G. Olmedo M. Qin X. (2012). Peroxiredoxins Are Conserved Markers of Circadian Rhythms. Nature 485 (7399), 459–464. 10.1038/nature11088 22622569 PMC3398137

[B25] Eide E. J. Vielhaber E. L. Hinz W. A. Virshup D. M. (2002). The Circadian Regulatory Proteins BMAL1 and Cryptochromes Are Substrates of Casein Kinase Iε. J. Biol. Chem. 277 (19), 17248–17254. 10.1074/jbc.M111466200 11875063 PMC1513548

[B26] Emery P. So W. V. Kaneko M. Hall J. C. Rosbash M. (1998). CRY, a Drosophila Clock and Light-Regulated Cryptochrome, Is a Major Contributor to Circadian Rhythm Resetting and Photosensitivity. Cell 95 (5), 669–679. 10.1016/s0092-8674(00)81637-2 9845369

[B27] Engel G. S. Calhoun T. R. Read E. L. Ahn T.-K. Mančal T. Cheng Y.-C. (2007). Evidence for Wavelike Energy Transfer through Quantum Coherence in Photosynthetic Systems. Nature 446 (7137), 782–786. 10.1038/nature05678 17429397

[B28] Falvey E. Fleury-Olela F. Schibler U. (1995). The Rat Hepatic Leukemia Factor (HLF) Gene Encodes Two Transcriptional Activators with Distinct Circadian Rhythms, Tissue Distributions and Target Preferences. EMBO J. 14 (17), 4307–4317. 10.1002/j.1460-2075.1995.tb00105.x 7556072 PMC394515

[B29] Foley L. E. Gegear R. J. Reppert S. M. (2011). Human Cryptochrome Exhibits Light-dependent Magnetosensitivity. Nat. Commun. 2, 356. 10.1038/ncomms1364 21694704 PMC3128388

[B30] Fonjallaz P. Ossipow V. Wanner G. Schibler U. (1996). The Two PAR Leucine Zipper Proteins, TEF and DBP, Display Similar Circadian and Tissue-specific Expression, but Have Different Target Promoter Preferences. EMBO J. 15 (2), 351–362. 10.1002/j.1460-2075.1996.tb00365.x 8617210 PMC449950

[B31] Fulco M. Cen Y. Zhao P. Hoffman E. P. McBurney M. W. Sauve A. A. (2008). Glucose Restriction Inhibits Skeletal Myoblast Differentiation by Activating SIRT1 through AMPK-Mediated Regulation of Nampt. Dev. cell 14 (5), 661–673. 10.1016/j.devcel.2008.02.004 18477450 PMC2431467

[B32] Gachon F. d. r. Nagoshi E. Brown S. Ripperger J. Schibler U. (2004). The Mammalian Circadian Timing System: From Gene Expression to Physiology. Chromosoma 113 (3), 103–112. 10.1007/s00412-004-0296-2 15338234

[B33] Gallego M. Virshup D. M. (2007). Post-translational Modifications Regulate the Ticking of the Circadian Clock. Nat. Rev. Mol. Cell Biol. 8 (2), 139–148. 10.1038/nrm2106 17245414

[B34] Gao P. Yoo S.-H. Lee K.-J. Rosensweig C. Takahashi J. S. Chen B. P. (2013). Phosphorylation of the Cryptochrome 1 C-Terminal Tail Regulates Circadian Period Length. J. Biol. Chem. 288 (49), 35277–35286. 10.1074/jbc.M113.509604 24158435 PMC3853276

[B35] Gaston K. J. Duffy J. P. Gaston S. Bennie J. Davies T. W. (2014). Human Alteration of Natural Light Cycles: Causes and Ecological Consequences. Oecologia 176 (4), 917–931. 10.1007/s00442-014-3088-2 25239105 PMC4226844

[B36] Gauger E. M. Rieper E. Morton J. J. L. Benjamin S. C. Vedral V. (2011). Sustained Quantum Coherence and Entanglement in the Avian Compass. Phys. Rev. Lett. 106 (4), 040503. 10.1103/PhysRevLett.106.040503 21405313

[B37] Gegear R. J. Casselman A. Waddell S. Reppert S. M. (2008). Cryptochrome Mediates Light-dependent Magnetosensitivity in Drosophila. Nature 454 (7207), 1014–1018. 10.1038/nature07183 18641630 PMC2559964

[B38] Genov N. Castellana S. Scholkmann F. Capocefalo D. Truglio M. Rosati J. (2019). A Multi-Layered Study on Harmonic Oscillations in Mammalian Genomics and Proteomics. Ijms 20 (18), 4585. 10.3390/ijms20184585 PMC6770795 31533246

[B39] Gooley J. J. Rajaratnam S. M. W. Brainard G. C. Kronauer R. E. Czeisler C. A. Lockley S. W. (2010). Spectral Responses of the Human Circadian System Depend on the Irradiance and Duration of Exposure to Light. Sci. Transl. Med. 2 (31), 31ra33. 10.1126/scitranslmed.3000741 PMC4414925 20463367

[B41] Guerra P. A. Gegear R. J. Reppert S. M. (2014). A Magnetic Compass Aids Monarch Butterfly Migration. Nat. Commun. 5, 4164. 10.1038/ncomms5164 24960099 PMC4090716

[B42] Halberg F. Bittner J. J. Gully R. J. Albrecht P. G. Brackney E. L. (1955). 24-Hour Periodicity and Audiogenic Convulsions in I Mice of Various Ages. Exp. Biol. Med. 88 (2), 169–173. Society for Experimental Biology and Medicine (New York, N.Y.). 10.3181/00379727-88-21526 14357377

[B43] Hardin P. E. Panda S. (2013). Circadian Timekeeping and Output Mechanisms in Animals. Curr. Opin. Neurobiol. 23 (5), 724–731. 10.1016/j.conb.2013.02.018 23731779 PMC3973145

[B44] Harmer S. L. Panda S. Kay S. A. (2001). Molecular Bases of Circadian Rhythms. Annu. Rev. Cell Dev. Biol. 17, 215–253. 10.1146/annurev.cellbio.17.1.215 11687489

[B45] Hayashi T. Stuchebrukhov A. A. (2010). Electron Tunneling in Respiratory Complex I. Proc. Natl. Acad. Sci. U.S.A. 107 (45), 19157–19162. 10.1073/pnas.1009181107 20974925 PMC2984193

[B47] Hiscock H. G. Worster S. Kattnig D. R. Steers C. Jin Y. Manolopoulos D. E. (2016). The Quantum Needle of the Avian Magnetic Compass. Proc. Natl. Acad. Sci. U.S.A. 113 (17), 4634–4639. 10.1073/pnas.1600341113 27044102 PMC4855607

[B48] Honma S. (2018). The Mammalian Circadian System: A Hierarchical Multi-Oscillator Structure for Generating Circadian Rhythm. J. Physiol. Sci. 68 (3), 207–219. 10.1007/s12576-018-0597-5 29460036 PMC10717972

[B49] Hore P. (2019). Upper Bound on the Biological Effects of 50/60 Hz Magnetic Fields Mediated by Radical Pairs. Elife 8, e44179. 10.7554/eLife.44179 30801245 PMC6417859

[B50] Ishizaki A. Fleming G. R. (2009). Theoretical Examination of Quantum Coherence in a Photosynthetic System at Physiological Temperature. Proc. Natl. Acad. Sci. U.S.A. 106 (41), 17255–17260. 10.1073/pnas.0908989106 19815512 PMC2762676

[B51] Iwasaki H. Nishiwaki T. Kitayama Y. Nakajima M. Kondo T. (2002). KaiA-stimulated KaiC Phosphorylation in Circadian Timing Loops in Cyanobacteria. Proc. Natl. Acad. Sci. U.S.A. 99 (24), 15788–15793. 10.1073/pnas.222467299 12391300 PMC137794

[B52] Jagannath A. Taylor L. Wakaf Z. Vasudevan S. R. Foster R. G. (2017). The Genetics of Circadian Rhythms, Sleep and Health. Hum. Mol. Genet. 26, R128–R138. 10.1093/hmg/ddx240 28977444 PMC5886477

[B53] Johnson C. H. (2004). Precise Circadian Clocks in Prokaryotic Cyanobacteria. Curr. Issues Mol. Biol. 6 (2), 103–110. 10.21775/cimb.006.103 15119821

[B54] Johnson C. H. (2007). Bacterial Circadian Programs. Cold Spring Harb. symposia quantitative Biol. 72, 395–404. 10.1101/sqb.2007.72.027 18419297

[B55] Johnson C. H. Mori T. Xu Y. (2008). A Cyanobacterial Circadian Clockwork. Curr. Biol. 18 (17), R816–R825. 10.1016/j.cub.2008.07.012 18786387 PMC2585598

[B56] Jordan S. D. Lamia K. A. (2013). AMPK at the Crossroads of Circadian Clocks and Metabolism. Mol. Cell. Endocrinol. 366 (2), 163–169. 10.1016/j.mce.2012.06.017 22750052 PMC3502724

[B57] Kang T.-H. (2021). Circadian Rhythm of NER and ATR Pathways. Biomolecules 11 (5), 715. 10.3390/biom11050715 34064641 PMC8150605

[B58] Kang T.-H. Sancar A. (2009). Circadian Regulation of DNA Excision Repair: Implications for Chrono-Chemotherapy. Cell Cycle 8 (11), 1665–1667. 10.4161/cc.8.11.8707 19411851

[B59] Klinman J. P. Kohen A. (2013). Hydrogen Tunneling Links Protein Dynamics to Enzyme Catalysis. Annu. Rev. Biochem. 82, 471–496. 10.1146/annurev-biochem-051710-133623 23746260 PMC4066974

[B60] Koike N. Yoo S.-H. Huang H.-C. Kumar V. Lee C. Kim T.-K. (2012). Transcriptional Architecture and Chromatin Landscape of the Core Circadian Clock in Mammals. Science 338 (6105), 349–354. 10.1126/science.1226339 22936566 PMC3694775

[B61] Konik R. (2021). Quantum Coherence Confined. Nat. Phys. 17, 669–670. 10.1038/s41567-021-01211-5

[B62] Kornmann B. Schaad O. Reinke H. Saini C. Schibler U. (2007). Regulation of Circadian Gene Expression in Liver by Systemic Signals and Hepatocyte Oscillators. Cold Spring Harb. symposia quantitative Biol. 72, 319–330. 10.1101/sqb.2007.72.041 18419289

[B63] Lambert N. Chen Y.-N. Cheng Y.-C. Li C.-M. Chen G.-Y. Nori F. (2013). Quantum Biology. Nat. Phys. 9, 10–18. 10.1038/nphys2474

[B64] Lamia K. A. Sachdeva U. M. DiTacchio L. Williams E. C. Alvarez J. G. Egan D. F. (2009). AMPK Regulates the Circadian Clock by Cryptochrome Phosphorylation and Degradation. Science 326 (5951), 437–440. 10.1126/science.1172156 19833968 PMC2819106

[B65] Lloyd S. (2011). Quantum Coherence in Biological Systems. J. Phys. Conf. Ser. 302, 012037. 10.1088/1742-6596/302/1/012037

[B66] Longuet-Higgins H. C. (1962). Quantum Mechanics and Biology. Biophysical J. 2, 207–215. 10.1016/s0006-3495(62)86957-4 PMC1366485 14466521

[B68] Löwdin P.-O. (1963). Proton Tunneling in DNA and its Biological Implications. Rev. Mod. Phys. 35, 724–732. 10.1103/RevModPhys.35.724

[B69] MacGregor-Chatwin C. Sener M. Barnett S. F. H. Hitchcock A. Barnhart-Dailey M. C. Maghlaoui K. (2017). Lateral Segregation of Photosystem I in Cyanobacterial Thylakoids. Plant Cell 29 (5), 1119–1136. 10.1105/tpc.17.00071 28364021 PMC5466035

[B70] Mailloux R. J. Jin X. Willmore W. G. (2014). Redox Regulation of Mitochondrial Function with Emphasis on Cysteine Oxidation Reactions. Redox Biol. 2, 123–139. 10.1016/j.redox.2013.12.011 24455476 PMC3895620

[B71] Marais A. Adams B. Ringsmuth A. K. Ferretti M. Gruber J. M. Hendrikx R. (2018). The Future of Quantum Biology. J. R. Soc. Interface. 15 (148), 20180640. 10.1098/rsif.2018.0640 30429265 PMC6283985

[B72] Mazzoccoli G. Miscio G. Fontana A. Copetti M. Francavilla M. Bosi A. (2016). Time Related Variations in Stem Cell Harvesting of Umbilical Cord Blood. Sci. Rep. 6, 21404. 10.1038/srep21404 26906327 PMC4764902

[B73] Mendoza J. (2007). Circadian Clocks: Setting Time by Food. J. Neuroendocrinol. 19 (2), 127–137. 10.1111/j.1365-2826.2006.01510.x 17214875

[B74] Mendoza J. (2019). Eating Rewards the Gears of the Clock. Trends Endocrinol. Metabolism 30 (5), 299–311. 10.1016/j.tem.2019.03.001 30935670

[B75] Mistlberger R. E. Antle M. C. (2011). Entrainment of Circadian Clocks in Mammals by Arousal and Food. Essays Biochem. 49 (1), 119–136. 10.1042/bse0490119 21819388

[B76] Mohawk J. A. Green C. B. Takahashi J. S. (2012). Central and Peripheral Circadian Clocks in Mammals. Annu. Rev. Neurosci. 35, 445–462. 10.1146/annurev-neuro-060909-153128 22483041 PMC3710582

[B77] Nagoshi E. Saini C. Bauer C. Laroche T. Naef F. Schibler U. (2004). Circadian Gene Expression in Individual Fibroblasts. Cell 119 (5), 693–705. 10.1016/j.cell.2004.11.015 15550250

[B78] Nakahata Y. Kaluzova M. Grimaldi B. Sahar S. Hirayama J. Chen D. (2008). The NAD+-dependent Deacetylase SIRT1 Modulates CLOCK-Mediated Chromatin Remodeling and Circadian Control. Cell 134 (2), 329–340. 10.1016/j.cell.2008.07.002 18662547 PMC3526943

[B79] Nakahata Y. Sahar S. Astarita G. Kaluzova M. Sassone-Corsi P. (2009). Circadian Control of the NAD + Salvage Pathway by CLOCK-SIRT1. Science 324, 654–657. 10.1126/science.1170803 19286518 PMC6501775

[B81] Neufeld-Cohen A. Robles M. S. Aviram R. Manella G. Adamovich Y. Ladeuix B. (2016). Circadian Control of Oscillations in Mitochondrial Rate-Limiting Enzymes and Nutrient Utilization by PERIOD Proteins. Proc. Natl. Acad. Sci. U.S.A. 113 (12), E1673–E1682. 10.1073/pnas.1519650113 26862173 PMC4812734

[B82] Noordally Z. B. Ishii K. Atkins K. A. Wetherill S. J. Kusakina J. Walton E. J. (2013). Circadian Control of Chloroplast Transcription by a Nuclear-Encoded Timing Signal. Science 339 (6125), 1316–1319. 10.1126/science.1230397 23493713

[B83] Ozturk N. (2021). Light‐dependent Reactions of Animal Circadian Photoreceptor Cryptochrome. FEBS J. 9. 10.1111/febs.16273 34750956

[B84] Panitchayangkoon G. Hayes D. Fransted K. A. Caram J. R. Harel E. Wen J. (2010). Long-lived Quantum Coherence in Photosynthetic Complexes at Physiological Temperature. Proc. Natl. Acad. Sci. U.S.A. 107 (29), 12766–12770. 10.1073/pnas.1005484107 20615985 PMC2919932

[B85] Peek C. B. Affinati A. H. Ramsey K. M. Kuo H.-Y. Yu W. Sena L. A. (2013). Circadian Clock NAD + Cycle Drives Mitochondrial Oxidative Metabolism in Mice. Science 342 (6158), 1243417. 10.1126/science.1243417 24051248 PMC3963134

[B86] Petrillo E. Godoy Herz M. A. Fuchs A. Reifer D. Fuller J. Yanovsky M. J. (2014). A Chloroplast Retrograde Signal Regulates Nuclear Alternative Splicing. Science 344 (6182), 427–430. 10.1126/science.1250322 24763593 PMC4382720

[B87] Pfeifer G. P. You Y.-H. Besaratinia A. (2005). Mutations Induced by Ultraviolet Light. Mutat. Research/Fundamental Mol. Mech. Mutagen. 571 (1-2), 19–31. 10.1016/j.mrfmmm.2004.06.057 15748635

[B88] Pinzon-Rodriguez A. Bensch S. Muheim R. (2018). Expression Patterns of Cryptochrome Genes in Avian Retina Suggest Involvement of Cry4 in Light-dependent Magnetoreception. J. R. Soc. Interface. 15 (140), 20180058. 10.1098/rsif.2018.0058 29593090 PMC5908540

[B89] Pusuluk O. Farrow T. Deliduman C. Burnett K. Vedral V. (2018). Proton Tunnelling in Hydrogen Bonds and its Implications in an Induced-Fit Model of Enzyme Catalysis. Proc. R. Soc. A 474, 20180037. 10.1098/rspa.2018.0037

[B90] Pusuluk O. Torun G. Deliduman C. (2018). Quantum Entanglement Shared in Hydrogen Bonds and its Usage as a Resource in Molecular Recognition. Mod. Phys. Lett. B 32, 1850308. 10.1142/S0217984918503086

[B91] Ramsey K. M. Yoshino J. Brace C. S. Abrassart D. Kobayashi Y. Marcheva B. (2009). Circadian Clock Feedback Cycle through NAMPT-Mediated NAD + Biosynthesis. Science 324, 651–654. 10.1126/science.1171641 19299583 PMC2738420

[B92] Rieper, E., Anders, J., and Vedral, V. (2010). The Relevance of Continuous Variable Entanglement in DNA. arXiv:1006.4053v1.Quantum Physics [quant-ph] https://doi.org/10.48550/arXiv.1006.4053

[B93] Ritz T. Thalau P. Phillips J. B. Wiltschko R. Wiltschko W. (2004). Resonance Effects Indicate a Radical-Pair Mechanism for Avian Magnetic Compass. Nature 429 (6988), 177–180. 10.1038/nature02534 15141211

[B94] Ritz T. Wiltschko R. Hore P. J. Rodgers C. T. Stapput K. Thalau P. (2009). Magnetic Compass of Birds Is Based on a Molecule with Optimal Directional Sensitivity. Biophysical J. 96 (8), 3451–3457. 10.1016/j.bpj.2008.11.072 PMC2718301 19383488

[B95] Ruan G.-X. Allen G. C. Yamazaki S. McMahon D. G. (2008). An Autonomous Circadian Clock in the Inner Mouse Retina Regulated by Dopamine and GABA. PLoS Biol. 6 (10), e249. 10.1371/journal.pbio.0060249 18959477 PMC2567003

[B96] Ruan G.-X. Zhang D.-Q. Zhou T. Yamazaki S. McMahon D. G. (2006). Circadian Organization of the Mammalian Retina. Proc. Natl. Acad. Sci. U.S.A. 103 (25), 9703–9708. 10.1073/pnas.0601940103 16766660 PMC1480470

[B97] Sahar S. Sassone-Corsi P. (2013). The Epigenetic Language of Circadian Clocks. Handb. Exp. Pharmacol. 2013(217), 29–44. 10.1007/978-3-642-25950-0_2 23604474

[B98] Sanchez S. E. Kay S. A. (2016). The Plant Circadian Clock: From a Simple Timekeeper to a Complex Developmental Manager. Cold Spring Harb. Perspect. Biol. 8 (12), a027748. 10.1101/cshperspect.a027748 27663772 PMC5131769

[B99] Schibler U. Gotic I. Saini C. Gos P. Curie T. Emmenegger Y. (2015). Clock-Talk: Interactions between Central and Peripheral Circadian Oscillators in Mammals. Cold Spring Harb. Symp. Quant. Biol. 80, 223–232. 10.1101/sqb.2015.80.027490 26683231

[B100] Scholkmann F. Miscio G. Tarquini R. Bosi A. Rubino R. di Mauro L. (2016). The Circadecadal Rhythm of Oscillation of Umbilical Cord Blood Parameters Correlates with Geomagnetic Activity - an Analysis of Long-Term Measurements (1999-2011). Chronobiology Int. 33 (9), 1136–1147. 10.1080/07420528.2016.1202264 27409251

[B101] Schrödinger E. (1944). What Is Life? the Physical Aspect of the Living Cell. The Edinburgh BuildingCambridge, UK: Cambridge University Press. First published.

[B102] Scrima R. Cela O. Agriesti F. Piccoli C. Tataranni T. Pacelli C. (2020). Mitochondrial Calcium Drives Clock Gene-dependent Activation of Pyruvate Dehydrogenase and of Oxidative Phosphorylation. Biochimica Biophysica Acta (BBA) - Mol. Cell Res. 1867 (11), 118815. 10.1016/j.bbamcr.2020.118815 32763264

[B103] Scrima R. Cela O. Merla G. Augello B. Rubino R. Quarato G. (2016). Clock-genes and Mitochondrial Respiratory Activity: Evidence of a Reciprocal Interplay. Biochimica Biophysica Acta (BBA) - Bioenergetics 1857 (8), 1344–1351. 10.1016/j.bbabio.2016.03.035 27060253

[B104] Sia P. I. Luiten A. N. Stace T. M. Wood J. P. Casson R. J. (2014). Quantum Biology of the Retina. Clin. Exp. Ophthalmol. 42 (6), 582–589. 10.1111/ceo.12373 24979043

[B105] Simpson M. G. (2019). “Introduction,” in Plant Systematics. Editor Simpson Michael. G.. Third Edition (Cambridge: Academic Press), 467. ISBN 9780128126288. 10.1016/B978-0-12-812628-8.50033-X

[B106] Srivastava R. (2019). The Role of Proton Transfer on Mutations. Front. Chem. 7, 536. 10.3389/fchem.2019.00536 31497591 PMC6712085

[B108] Takahashi J. S. Hong H.-K. Ko C. H. McDearmon E. L. (2008). The Genetics of Mammalian Circadian Order and Disorder: Implications for Physiology and Disease. Nat. Rev. Genet. 9 (10), 764–775. 10.1038/nrg2430 18802415 PMC3758473

[B109] Takahashi J. S. (2017). Transcriptional Architecture of the Mammalian Circadian Clock. Nat. Rev. Genet. 18 (3), 164–179. 10.1038/nrg.2016.150 27990019 PMC5501165

[B110] Tarquini R. Mazzoccoli G. (2017). Clock Genes, Metabolism, and Cardiovascular Risk. Heart Fail. Clin. 13 (4), 645–655. 10.1016/j.hfc.2017.05.001 28865774

[B111] Thompson C. L. Sancar A. (2002). Photolyase/cryptochrome Blue-Light Photoreceptors Use Photon Energy to Repair DNA and Reset the Circadian Clock. Oncogene 21 (58), 9043–9056. 10.1038/sj.onc.1205958 12483519

[B112] Thyrhaug E. Tempelaar R. Alcocer M. J. P. Žídek K. Bína D. Knoester J. (2018). Identification and Characterization of Diverse Coherences in the Fenna-Matthews-Olson Complex. Nat. Chem. 10 (7), 780–786. 10.1038/s41557-018-0060-5 29785033

[B113] Tosini G. Davidson A. J. Fukuhara C. Kasamatsu M. Castanon‐Cervantes O. (2007). Localization of a Circadian Clock in Mammalian Photoreceptors. FASEB J. 21 (14), 3866–3871. 10.1096/fj.07-8371com 17621597 PMC2385786

[B114] Ukai H. Ueda H. R. (2010). Systems Biology of Mammalian Circadian Clocks. Annu. Rev. Physiol. 72, 579–603. 10.1146/annurev-physiol-073109-130051 20148689

[B115] Varn D. P. Crutchfield J. P. (2016). What Did Erwin Mean? the Physics of Information from the Materials Genomics of Aperiodic Crystals and Water to Molecular Information Catalysts and Life. Phil. Trans. R. Soc. A 374 (2063), 20150067. 10.1098/rsta.2015.0067 26857672

[B116] Visscher M. B. Halberg F. (1955). Daily Rhythms in Numbers of Circulating Eosinophils and Some Related Phenomena. Ann. N. Y. Acad. Sci. 59 (5), 834–849. 10.1111/j.1749-6632.1955.tb45986.x 13259354

[B117] Wan G. Hayden A. N. Iiams S. E. Merlin C. (2021). Cryptochrome 1 Mediates Light-dependent Inclination Magnetosensing in Monarch Butterflies. Nat. Commun. 12 (1), 771. 10.1038/s41467-021-21002-z 33536422 PMC7859408

[B118] Warrant E. J. (2021). Unravelling the Enigma of Bird Magnetoreception. Nature 594 (7864), 497–498. 10.1038/d41586-021-01596-6 34163048

[B119] Wiltschko R. Nießner C. Wiltschko W. (2021). The Magnetic Compass of Birds: The Role of Cryptochrome. Front. Physiol. 12, 667000. 10.3389/fphys.2021.667000 34093230 PMC8171495

[B120] Wiltschko R. Wiltschko W. (2014). Sensing Magnetic Directions in Birds: Radical Pair Processes Involving Cryptochrome. Biosensors 4 (3), 221–242. 10.3390/bios4030221 25587420 PMC4264356

[B121] Woelfle M. A. Xu Y. Qin X. Johnson C. H. (2007). Circadian Rhythms of Superhelical Status of DNA in Cyanobacteria. Proc. Natl. Acad. Sci. U.S.A. 104 (47), 18819–18824. 10.1073/pnas.0706069104 18000054 PMC2141860

[B122] Wolynes P. G. (2009). Some Quantum Weirdness in Physiology. Proc. Natl. Acad. Sci. U.S.A. 106 (41), 17247–17248. 10.1073/pnas.0909421106 19815521 PMC2762665

[B123] Wong J. C. Y. Smyllie N. J. Banks G. T. Pothecary C. A. Barnard A. R. Maywood E. S. (2018). Differential Roles for Cryptochromes in the Mammalian Retinal Clock. FASEB J. 32 (8), 4302–4314. 10.1096/fj.201701165RR 29561690 PMC6071063

[B124] Xu J. Jarocha L. E. Zollitsch T. Konowalczyk M. Henbest K. B. Richert S. (2021). Magnetic Sensitivity of Cryptochrome 4 from a Migratory Songbird. Nature 594 (7864), 535–540. 10.1038/s41586-021-03618-9 34163056

[B125] Xu Y. Mori T. Johnson C. H. (2003). Cyanobacterial Circadian Clockwork: Roles of KaiA, KaiB and the kaiBC Promoter in Regulating KaiC. EMBO J. 22 (9), 2117–2126. 10.1093/emboj/cdg168 12727878 PMC156062

[B126] Yamaguchi S. Mitsui S. Yan L. Yagita K. Miyake S. Okamura H. (2000). Role of DBP in the Circadian Oscillatory Mechanism. Mol. Cell Biol. 20 (13), 4773–4781. 10.1128/MCB.20.13.4773-4781.2000 10848603 PMC85912

[B128] Ye R. Selby C. P. Chiou Y.-Y. Ozkan-Dagliyan I. Gaddameedhi S. Sancar A. (2014). Dual Modes of CLOCK:BMAL1 Inhibition Mediated by Cryptochrome and Period Proteins in the Mammalian Circadian Clock. Genes Dev. 28 (18), 1989–1998. 10.1101/gad.249417.114 25228643 PMC4173159

